# A Silenced *vanA* Gene Cluster on a Transferable Plasmid Caused an Outbreak of Vancomycin-Variable Enterococci

**DOI:** 10.1128/AAC.00286-16

**Published:** 2016-06-20

**Authors:** Audun Sivertsen, Torunn Pedersen, Kjersti Wik Larssen, Kåre Bergh, Torunn Gresdal Rønning, Andreas Radtke, Kristin Hegstad

**Affiliations:** a Research Group for Host-Microbe Interactions, Department of Medical Biology, Faculty of Health Sciences, University of Tromsø—The Arctic University of Norway, Tromsø, Norway; b Norwegian National Advisory Unit on Detection of Antimicrobial Resistance, Department of Microbiology and Infection Control, University Hospital of North-Norway, Tromsø, Norway; c Department of Medical Microbiology, St. Olavs University Hospital, Trondheim, Norway; d Unit for Infection Control, St. Olavs University Hospital, Trondheim, Norway; e Department of Laboratory Medicine, Children's and Women's Health, Norwegian University of Science and Technology, Trondheim, Norway

## Abstract

We report an outbreak of vancomycin-variable *vanA*^+^ enterococci (VVE) able to escape phenotypic detection by current guidelines and demonstrate the molecular mechanisms for *in vivo* switching into vancomycin resistance and horizontal spread of the *vanA* cluster. Forty-eight *vanA*^+^ Enterococcus faecium isolates and one Enterococcus faecalis isolate were analyzed for clonality with pulsed-field gel electrophoresis (PFGE), and their *vanA* gene cluster compositions were assessed by PCR and whole-genome sequencing of six isolates. The susceptible VVE strains were cultivated in brain heart infusion broth containing vancomycin at 8 μg/ml for *in vitro* development of resistant VVE. The transcription profiles of susceptible VVE and their resistant revertants were assessed using quantitative reverse transcription-PCR. Plasmid content was analyzed with S1 nuclease PFGE and hybridizations. Conjugative transfer of *vanA* was assessed by filter mating. The only genetic difference between the *vanA* clusters of susceptible and resistant VVE was an IS*L3*-family element upstream of *vanHAX*, which silenced *vanHAX* gene transcription in susceptible VVE. Furthermore, the VVE had an insertion of IS*1542* between *orf2* and *vanR* that attenuated the expression of *vanHAX*. Growth of susceptible VVE occurred after 24 to 72 h of exposure to vancomycin due to excision of the IS*L3*-family element. The *vanA* gene cluster was located on a transferable broad-host-range plasmid also detected in outbreak isolates with different pulsotypes, including one E. faecalis isolate. Horizontally transferable silenced *vanA* able to escape detection and revert into resistance during vancomycin therapy represents a new challenge in the clinic. Genotypic testing of invasive vancomycin-susceptible enterococci by *vanA*-PCR is advised.

## INTRODUCTION

The enterococci have adapted from harmless commensals to multiresistant nosocomial pathogens during the last decades (1). They may cause septicemia, urinary tract infections, endocarditis, and infection in indwelling catheters, predominantly as opportunistic infections (2, 3). In Enterococcus faecium, increased pathogenicity is explained by an expansion of hospital-adapted genetic lineages showing more resistance and virulence traits compared to commensal enterococci. Such traits are often encoded by mobile elements, which seem to accumulate in these lineages (4–6). Since ampicillin resistance in E. faecium is almost ubiquitous due to presence of multiple resistance determinants (3, 7) and gentamicin resistance is abundant (7, 8), treatment of E. faecium infections relies on the use of vancomycin. Resistance toward vancomycin is increasing worldwide (9), and the Scandinavian countries have experienced several dispersed vancomycin-resistant Enterococcus (VRE) outbreaks during the last years (10, 11), even though resistance rates are still low (7).

A total of eight gene clusters—*vanA*, *vanB*, *vanD*, *vanE*, *vanG*, *vanL*, *vanM*, and *vanN*—have been associated with acquired vancomycin resistance in enterococci (12–15). VanA, the most abundant resistance mechanism, confers high-level resistance by substituting the glycopeptide binding site in the peptidoglycan precursor termini from d-alanine to d-lactate by VanH, VanA, and VanX activities (16, 17). This system is regulated by VanS during glycopeptide exposure by phosphorylation and subsequent attachment of the VanR activator to specific upstream regions of the of *vanRS* and *vanHAX* promoters (16, 18, 19). Two accessory proteins depleting the cell wall of late peptidoglycan precursors containing a d-alanine residue (VanY) (20) and involved in low-level teicoplanin resistance by an unknown mechanism (VanZ) (21) are also included. The *vanA* gene cluster is normally associated with Tn*1546* (22).

As reported from several groups, the *vanA* gene cluster is prone to IS-element mediated alterations with occasional effects on vancomycin resistance phenotype, leading to phenotypes resembling VanB or VanD, as well as glycopeptide susceptibility (23–28). Leaving *vanA*^+^ VRE to grow in antibiotic-free media over a few months resulted in *in vitro* IS-element-mediated rearrangements of the *vanA* gene cluster, suggesting that rearrangements might be a common phenomenon (29). An outbreak of vancomycin susceptible enterococci containing *vanA* and capable of converting into a glycopeptide-resistant phenotype was recently reported in Canada. Such strains were termed vancomycin-variable enterococci (VVE) due to this ability (30, 31).

In July 2013 and January 2014, two patients from different wards of a Norwegian University Hospital were infected with vancomycin-susceptible E. faecium. After an ineffective course of vancomycin treatment, vancomycin-resistant E. faecium were isolated from the same wound of the first patient and a new blood culture of the second patient. Retesting of the initial isolates confirmed phenotypic susceptibility to vancomycin but revealed a *vanA* genotype. A prolonged screening program was initiated after confirmation of clonality for these four isolates, as well as two additional isolates from December 2013. We subsequently characterized 49 VVE and showed how deletion of an IS-element present in the *vanA* gene cluster rapidly altered the susceptible phenotype once the isolates were challenged with vancomycin. We also showed that *vanA* genes were located on a transferable broad-host-range plasmid that had spread the *vanA* gene cluster among unrelated E. faecium isolates and E. faecalis.

## MATERIALS AND METHODS

### Outbreak.

The initial two VVE isolates from cases 1 and 2 (Case1VVE-S and Case2VVE-S) were determined to be vancomycin susceptible according to EUCAST (European Committee on Antimicrobial Susceptibility Testing) disk diffusion analysis (using a 5-μg vancomycin disk on Muller-Hinton [MH] agar; Becton Dickinson [BBL], Sparks, MD), as well as Clinical and Laboratory Standards Institute screening (using 6 μg of vancomycin/ml in brain heart infusion [BHI] agar; Difco/Becton Dickinson) but were determined to be PCR positive for the *vanA* gene. The susceptible VVE isolates (VVE-S) did not grow on CHROMagar VRE (CHROMagar, Paris, France), whereas the resistant VVE (VVE-R; Case1VVE-R and Case2VVE-R) grew with pink colonies after 1 or 2 days. According to pulsed-field gel electrophoresis (PFGE), the four isolates were determined to be identical and, as determined by multilocus sequence typing (MLST), belonged to sequence type 203 (ST203). The two patients had been treated at different wards in separate buildings, but had both been admitted to St. Olavs University Hospital on several occasions between July 2013 and January 2014. Both had received vancomycin therapy for approximately 1 week between the isolation of VVE-S and VVE-R.

From January 2014 until 3 July 2015, 15,158 samples from 8,717 different patients, of which 14,883 screening samples and 275 clinical vancomycin-susceptible E. faecium isolates, were screened for the *vanA* containing vancomycin-variable E. faecium (VVE) genotype. All samples were analyzed by *vanA*-PCR, and 93 (0.61%) were positive. The numbers of positive screening tests by sample origin are shown in Table S1 in the supplemental material, along with further explanation of how included isolates were derived from screening in a flow diagram (see Fig. S1 in the supplemental material). In 57 of 93 cases, *vanA*^*+*^ enterococci could be isolated from feces and/or infected sites, in patients residing at 23 different wards. Of these 57 isolates, 3 were patient duplicates that did not change vancomycin resistance phenotype and were thus not included in this study. Five other isolates were not included by reasons indicated in Fig. S1 in the supplemental material. One of the VVE-S isolates obtained from rectal swab screenings appeared to be a *vanA*-PCR-positive E. faecalis.

### Clinical and screening sample processing.

Clinical samples were cultured on the department's conventional media according to sample type (see the methods note in the supplemental material for details). Screening samples, mainly rectal flocked swabs (Eswab; Copan) containing visible feces or feces in sterile containers, determined to be positive for *vanA* by PCR, were cultured on Enterococcosel agar (BBL) supplemented with ampicillin at 8 μg/ml and on CHROMagar VRE.

### Genomic DNA preparation from screening and clinical samples.

A 20-μl screening sample (Eswab or dissolved feces) was suspended in 200 μl of Tris-EDTA (TE) buffer and 20 μl of lysozyme (20 mg/ml; Sigma-Aldrich Corporation, St. Louis, MO). Alternatively, a single colony of E. faecium from clinical specimens was suspended in 200 μl of TE buffer, 20 μl of lysozyme, and 5 μl of proteinase K (20 mg/ml; Qiagen, Hilden, Germany). Screening samples were incubated for 10 min at room temperature; colonies were incubated in a thermomixer for 15 min at 37°C and at 65°C for 15 min. DNA was extracted on NucliSens easyMAG (bioMérieux, Marcy-l'Étoile, France).

### *vanA* PCR.

Rectal swabs and stool samples dissolved in 1 ml 0.9% NaCl were screened for *vanA* by an in-house real-time PCR targeting the *vanA* gene, with primers as described by Woodford et al. (32). From clinical specimens, a single colony identified as E. faecium by matrix-assisted laser desorption ionization–time of flight mass spectrometry was picked from blood agar.

Prior to the outbreak, a real-time PCR using EvaGreen and post-PCR melting analysis for verification of *vanA* on bacterial colonies had been established and was validated on stool specimens. Due to the large number of analyses, a TaqMan probe was designed after sequencing the PCR product of the initial six VVE outbreak isolates and CCUG59167. The primer and probe sequences are shown in Table S2 in the supplemental material. In addition all PCR-positive (stool specimens and) isolates were analyzed by the commercially available Xpert *vanA*/*vanB* assay (Cepheid, Sunnyvale, CA) to confirm presence of *vanA* by an alternative method.

PCR was performed on a CFX96 real-time PCR detection system (Bio-Rad) using the following reagents and conditions: 300 nM concentrations of each primer (*vanA*F and *vanA*R), a 200 nM concentration of *vanA* TaqMan probe (TIB Molbiol, Berlin, Germany), 10 μl of Perfecta Multiplex qPCR SuperMix UNG (Quanta BioSciences, Gaithersburg, MD), 3.5 μl of MG-water, and 5 μl of template (extracted genomic DNA). The two-step PCR protocol used was as follows: 45°C for 5 min, 95°C for 3 min, and then 40 cycles of 95°C for 5 s and 58°C for 30 s. Enterococcus faecium CCUG 59167 and water were used as positive and negative controls, respectively.

### Susceptibility testing.

Susceptibility testing of cultured enterococci was performed by the disk diffusion method for ampicillin, linezolid, and tigecycline by the EUCAST method on Mueller-Hinton agar (BBL) and interpreted using EUCAST breakpoints. Vancomycin resistance was screened for using BHI agar (Difco, Becton Dickinson) containing 6 μg of vancomycin/ml as recommended by Nordicast (33). Isolates displaying vancomycin resistance were confirmed by *vanA* PCR, and the level of vancomycin resistance was determined by vancomycin MIC test strips (Liofilchem, Roseto degli Abruzzi, Italy) according to the manufacturer's instructions.

After *in vitro* resistant mutant development, susceptibility testing of vancomycin and teicoplanin was done with MIC gradient strips (Liofilchem) and phenotypic resistance interpretation was performed according to EUCAST guidelines.

### PFGE and MLST.

PFGE was performed as described by Bannerman et al. (34) with slight modifications (see the methods for PFGE conditions and reagents in the supplemental material). Images were analyzed with BioNumerics software version 7.1.1 (Applied Maths, Sint-Martens-Latem, Belgium) with the Dice coefficient with a band position tolerance of 2.0% and an optimization of 1.5%. Cluster analysis was performed using unweighted pair group method with arithmetic averages (UPGMA). PFGE was interpreted according to the criteria of Tenover et al. (35). The MLST scheme developed for E. faecium was used according to previously published instructions on sequenced isolates (36).

### Whole-genome sequencing (WGS) and WGS comparison.

Four isolates collected from two patients before and after vancomycin treatment (Case1VVE-S, Case1VVE-R, Case2VVE-S, and Case2VVE-R) and two isolates from the screening period (Screen1VVE-S and Screen2VVE-S) were sequenced using Illumina MiSeq on 250-bp paired-end runs according to standard protocols. Raw reads were trimmed with EA-Utils (https://code.google.com/p/ea-utils) and processed through multiple assemblers in competition within the iMetAMOS pipeline v.1.5rc3 (37). SPAdes v.3.0.0 (38) produced the optimal assembly in all cases. Contigs smaller than 200 bp and with <2-fold coverage were removed by using an in-house script. Sequence data are available as BioProject PRJNA306646, and reads are available in the Short Read Archive under the Biosample accession numbers presented in Table S3 in the supplemental material.

In order to assign our WGS outbreak isolates into the Lebreton et al. data set (4), all genomes were downloaded and whole-genome aligned using the Harvest suite version 1.2 (39) with recombination filtration and forced inclusion of all isolates enabled. The phylogeny was created with Fasttree 2 (40), also included in the Harvest suite package and later edited by FigTree (http://tree.bio.ed.ac.uk/software/figtree/).

### *vanA* cluster and plasmid backbone characterization.

The in-house made PCRs *orf2-vanR*, *vanRS*, *vanSH*, *vanHAX*, *vanXY*, and *vanYZ* were performed on WGS isolate genomic DNA (gDNA) with primers as noted in Table S2 in the supplemental material. PCR products larger or smaller than the positive control BM4147 containing Tn*1546* without IS-element insertions were Sanger sequenced using BigDye 3.1 technology (Applied Biosystems, Waltham, MA). For E. faecium isolates considered identical to outbreak strain by PFGE (*n* = 42), Sanger sequencing of *vanA* cluster PCR products was not performed since similarity to WGS isolates was assumed. Linkage of the *vanA* cluster to the plasmid backbone in the E. faecalis isolate and E. faecium outbreak isolates with unique pulsotypes was performed using primers pVVE1-6F/R, as shown in Table S2 in the supplemental material.

### Switch from glycopeptide susceptibility to resistance.

Vancomycin resistance development was initiated by incubating a single susceptible VVE colony in 5 ml of BHI broth (Oxoid, Basingstoke, United Kingdom) overnight, followed by a 1:100 dilution into 5 ml of BHI broth containing 2 or 8 μg of vancomycin or teicoplanin/ml. With observation of growth every 12 h the first 2 days and every 24 h thereafter, emerged resistant mutants were diluted 10^6^-fold and plated on BHI agar containing 8 μg/ml vancomycin to obtain single colonies. All incubations were performed at 37°C. The *vanA* cluster structures of revertants were assessed by PCRs as described above.

### RNA extraction and quantitative reverse transcription-PCR (RT-qPCR).

E. faecium Case1VVE-S and the *in vitro*-generated vancomycin-resistant mutant, as well as control BM4147, were grown in 15 ml of BHI while recording medium turbidity with a spectrophotometer. Total RNA was extracted from 2 ml of mid-log-phase cultures by using an RNeasy Protect bacterial minikit (Qiagen) according to the manufacturer's instructions with 20,000 U of mutanolysin (Sigma-Aldrich) added to the lysis step. Contaminating DNA was removed by using the Heat&Run gDNA removal kit (Arcticzymes, Tromsø, Norway) and cDNA produced from 100 ng of RNA by using the high-capacity RNA-to-cDNA kit (Applied Biosystems) according to the manufacturer's instructions. Primers and TaqMan probes for real-time PCRs are listed in Table S2 in the supplemental material, and PCR was performed using qPCR Mastermix Plus Low ROX (Eurogentec, Liege, Belgium) according to standard protocols supplied by the manufacturer. Reactions without reverse transcriptase were used as a control for DNA contamination after DNase treatment. All qPCRs were performed in triplicates. ΔRn threshold was standardized for all reactions. The Livak method was used to calculate the fold changes (41).

### *In vitro* horizontal transfer of plasmid.

Filter mating and subsequent verification of transconjugants using SmaI restriction PFGE, as well as S1 nuclease restriction PFGE, followed by Southern hybridization, were performed as described by Sivertsen et al. (10). We conducted two experiments using either vancomycin (8 μg/ml) or chloramphenicol (8 μg/ml) as a selective agent. Filter-mated bacteria were cultured on BHI agar plates containing either (i) one of the selective antibiotics, (ii) rifampin (20 μg/ml) and fusidic acid (10 μg/ml), or (iii) vancomycin or chloramphenicol combined with rifampin and fusidic acid (ii). The primers used to produce probes for Southern hybridization are given in Table S2 in the supplemental material.

## RESULTS

### Extended screening efforts show wide dispersal of clonal VVE in several wards.

**FIG 1:**
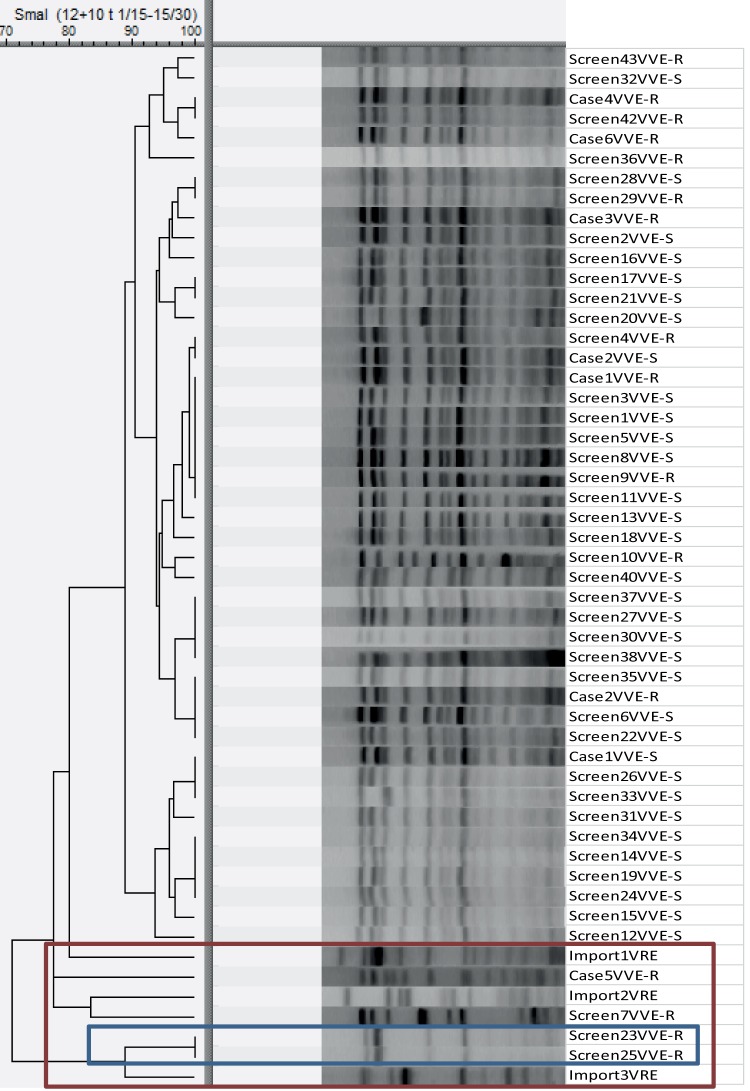
PFGE comparison of VVE E. faecium and VRE E. faecium isolated during this outbreak, with a UPGMA tree illustrating the distance between isolates. Inside the red box are pulsotypes of all isolates regarded unrelated to the main cluster. The blue box shows a local cluster of unrelated VVE within one single ward.

SmaI restriction PFGE (Fig. 1) is shown for 52 identified *vanA*^*+*^ E. faecium, including the two index cases and subsequent clinical and screening isolates. PFGE clustering showed a dominant E. faecium clone (*n* = 45) found primarily as a colonizer in hospital admitted patients. We found four isolates with three unique PFGE types dissimilar to the outbreak clone in patients colonized (Screen7VVE-R, Screen23VVE-R, and Screen25VVE-R) or infected (Case5VVE-R) with E. faecium. Lastly, a *vanA*-carrying susceptible E. faecalis isolate (Screen41VVEfs-S) was included in the study to investigate a possible linkage to the VVE faecium. Demographic data, antibiograms, and analysis results of all included isolates (*n* = 49) can be found in Table S3 in the supplemental material. MLST data extracted from WGS of six VVE showed that they belonged to ST203.

### Difference in composition of the *vanA* gene cluster in susceptible and resistant isolates.

**FIG 2:**
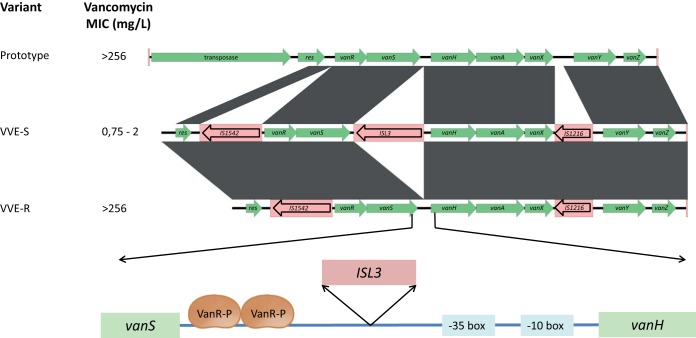
Insertion site of I*SL3* illustrated in a scaled alignment of *vanA* clusters from Norwegian clonal VVE-S and VVE-R to prototype Tn*1546* (GenBank accession no. M97297). In the zoomed view, the location of I*SL3* between the binding site of the VanR activator (VanR-P) and the *vanHAX* promoter (−35 and −10 boxes) is indicated.

All six sequenced isolates contained the *vanA* gene cluster, although in contigs which had to be joined by gap closure PCR and Sanger sequencing of PCR products of intergenic regions between *orf2* and *vanR*, *vanS*, and *vanH* and between *vanX* and *vanY*. Compared to the prototypic Tn*1546* (GenBank accession no. M97297), an IS*L3*-family element was inserted between the VanR binding site and the *vanHAX* promoter region in susceptible VVE isolates (Fig. 2). IS*L3* was absent in both Case1VVE-R and Case2VVE-R which otherwise showed a *vanA* cluster identical to the VVE-S isolates.

Case1VVE-S, Case2VVE-S, Screen1VVE-S, and Screen2VVE-S had IS*1542*, IS*L3*, and IS*1216V* inserted at positions 3924 to 3933, 4977, and 8649 to 8832, respectively, as indicated in Fig. 2, with IS*1542* and IS*1216V* insertions causing deletion of 9 and 183 bases, respectively. The transposase and part of the resolvase constituting the Tn*1546* transposition machinery were missing from all six isolates due to a deletion upstream of position 3419.

### Switch from vancomycin susceptibility to resistance during selection by IS*L3* excision.

Loss of the IS*L3* element upstream of the *vanHAX* operon is a plausible reason for phenotypic shift to vancomycin resistance in the otherwise isogenic clinical isolates. To investigate this, the phenotypically susceptible Case1VVE-S, Case2VVE-S, Screen1VVE-S, and Screen2VVE-S isolates were cultured in the presence of vancomycin or teicoplanin either slightly above (8 μg/ml) the EUCAST clinical breakpoints (vancomycin resistant (R) > 4 μg/ml; teicoplanin R > 2 μg/ml) or just under (2 μg/ml).

During 8-μg/ml vancomycin exposure, three of the four isolates exerted a prolonged lag phase with growth occurring after 24 to 48 h. PCR analyses of the *in vitro* revertants revealed restoration of the promoter/activator binding region of *vanHAX* by IS*L3* loss. In the fourth isolate, no growth could be seen during the 7 days the experiment lasted. The phenotype of revertants obtained was confirmed by MIC test strip analyses that showed high-level resistance toward both vancomycin and teicoplanin. Subsequent exposure of all susceptible *vanA*^*+*^ isolates recovered during the screening period (Screen3-41VVE) to vancomycin at 8 μg/ml showed that 30 of 31 reverted to the resistant phenotype after 1 to 5 days. PCR analyses of the resulting revertants indeed showed IS*L3* loss in all cases (see Table S3 in the supplemental material). We also exposed the six sequenced isolates to teicoplanin at 8 μg/ml and similarly obtained a phenotypic switch caused by IS*L3* loss (data not shown).

When the WGS isolates were subjected to vancomycin in 2-μg/ml concentrations, the growth lag varied from 24 to 148 h (12 days), and several *vanA* gene cluster variations were observed in the revertants. PCR analyses and DNA sequencing revealed the deletion of *vanX* and *vanY* and a deletion in the *vanSH* intergenic region in some revertants. The gene cluster variations arising by sub-MIC exposure of vancomycin resulted in decreased teicoplanin MIC in two of three cases and in one case also gave low-level vancomycin resistance (see Table S3 in the supplemental material).

### IS elements perturb transcription of *vanHAX* and *vanRS*.

**FIG 3:**
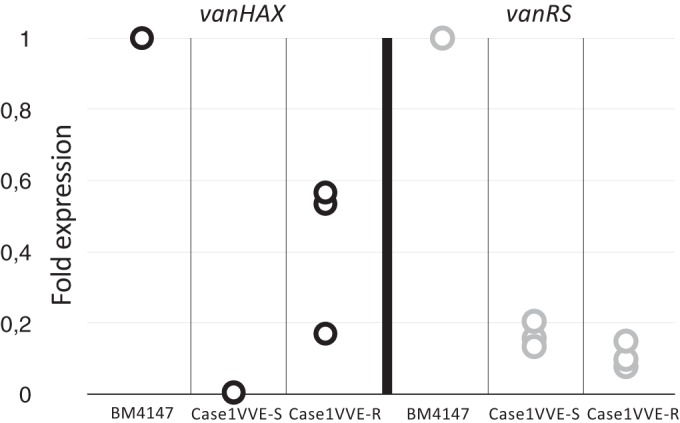
Expression levels of the *vanHAX* and *vanRS* operons in the VanA-silenced (Case1VVE-S) and resistant (Case1VVE-R) isolates relative to BM4147 (Tn*1546* prototype) assessed by RT-qPCR. Data points for three independent experiments are shown for each isolate. All measurements were normalized against the housekeeping gene glutamate dehydrogenase (*gdh*).

We hypothesized that the IS*1542* and IS*L3* insertions influenced expression of the two operons regarded essential for the VanA phenotype, *vanHAX* and *vanRS*. Transcription levels of the *vanHAX* and *vanRS* operons were analyzed by RT-qPCR comparing the Tn*1546* prototype strain BM4147, Case1VVE-S and Case1VVE-R. Figure 3 shows the relative expression of *vanRS* and *vanHAX* in the susceptible and resistant isolates by using expression in BM4147 as a calibrator and *gdh* as an endogenous control for normalization. IS*L3* insertion leads to silencing of the *vanHAX* operon, as demonstrated by comparing Case1VVE-S (ΔΔ*C_T_* = 0.004 to 0.005) to Case1VVE-R (ΔΔ*C_T_* = 0.16 to 0.53) grown in BHI.

In Case1VVE-S and Case1VVE-R, the introduction of IS*1542* upstream of *vanRS* leads to attenuated *vanRS* expression (ΔΔ*C_T_* = 0.08 to 0.20). Accordingly, the observed expression of *vanHAX* was reduced in Case1VVE-R relative to BM4147 (encoding the Tn*1546* prototype).

### The *vanA* gene cluster is located on a transferable broad-host-range plasmid.

Examination of a 10-kb stretch of the assembled DNA downstream of *vanXY* showed high homology to plasmids of the broad-host-range Inc18 family (42), most extensively to the pRE25 plasmid of E. faecalis (43). Moreover, the presence of a replication initiation gene (*rep*) of replicon class 1 represented by reference plasmid pIP501 of Streptococcus agalactiae (44), rendered a plasmid linkage of the *vanA* cluster probable. A *cat* chloramphenicol resistance determinant was also colocated in this region. Interestingly, PCR analyses linked the *vanA* gene cluster of an E. faecalis strain isolated as part of the outbreak screening to the same 10-kb stretch downstream of *vanXY*. Linkage was similarly also found in five E. faecium not related to the outbreak clone by PFGE typing. The five genetically unrelated E. faecium and the E. faecalis isolate possessed IS-element insertions in their *vanA* gene cluster similar to those of the outbreak isolates. Taken together, this suggests horizontal transfer of a mobile element containing this particular *vanA* cluster variant.

**FIG 4:**
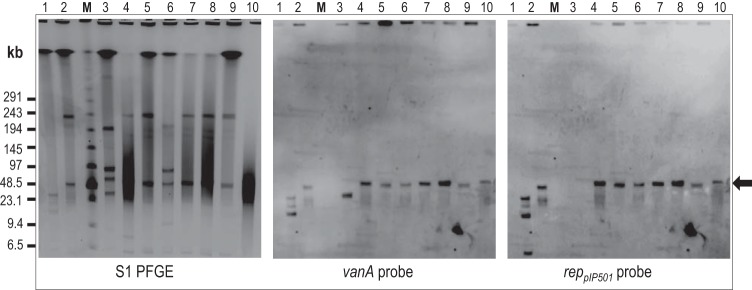
Plasmid profiles of E. faecium outbreak isolates including pulsotypes unrelated to the main clone and the E. faecalis isolate, as shown by S1 nuclease restriction PFGE and subsequent Southern blotting and hybridization with indicated probes. Lanes: 1, BM4147 *vanA*^*+*^ control; 2, Case1VVE-S; 3, *rep*_pIP*501*_ control; 4, Case5VVE-R; 5, Screen7VVE-R; 6, Screen10VVE-R; 7, Screen23VVE-R; 8, Screen25VVE-R; 9, Screen38VVE-S; 10, Screen41VVE-S (E. faecalis). The sizes of the molecular marker (M) are indicated.

To investigate such plasmid linkage, as well as the transferability of the *vanA* gene cluster from the outbreak isolates, cohybridization and *in vitro* filter-mating analyses were performed. Plasmid profiling of the four unrelated outbreak E. faecium isolates and the *vanA*^*+*^ E. faecalis isolate was conducted by S1 nuclease restriction and PFGE. The subsequent Southern hybridization with *vanA* and *rep*_pIP*501*_ probes revealed presence of a plasmid with a size of ∼50 kb that harbored the *vanA* gene cluster and cohybridized with a *rep*_pIP*501*_ probe (Fig. 4) in all the outbreak related isolates.

We achieved *in vitro* horizontal transfer of the *vanA* gene cluster by selective pressure of either vancomycin or chloramphenicol with Case1VVE-R or Case1VVE-S as donors and plasmid-free strain 64/3 as a recipient. The presence of the plasmid in transconjugants was confirmed by S1 nuclease restriction, as well as by cohybridization analyses using *vanA* and *rep*_pIP*501*_ probes (see Fig. S2B in the supplemental material). Horizontal gene transfer from donors to the recipient strain was confirmed by SmaI restriction PFGE of three collected transconjugants per filter mating (see Fig. S2A in the supplemental material). Transfer occurred with a frequency of 9 × 10^−5^ (Case1VVE-R) transconjugants/donor with chloramphenicol selection and 1 × 10^−7^ (Case1VVE-R) transconjugants/donor during vancomycin selection.

### Global epidemiological linkage of the VVE clone.

To illustrate the clade specificity of the ST203 outbreak clone, a core-genome alignment phylogeny was generated by parsnp v.1.2 (39) (see Fig. S3 in the supplemental material) including the six WGS isolates from this study as well as isolates previously analyzed by Lebreton et al. (4) The six VVE cluster with other ST203 isolates.

## DISCUSSION

The term VVE should be restricted to vancomycin-susceptible enterococci containing *vanA* and capable of reverting to a glycopeptide-resistant phenotype. Accordingly, enterococci containing remnants of the *vanA* cluster that are not able to revert to a resistant phenotype or enterococci with *vanB* showing an MIC below the clinical breakpoint are not VVE.

We disclose here the molecular characteristics of enterococci isolated during an outbreak of vancomycin-susceptible, *vanA*-positive enterococci in Norway. To our knowledge, the first occurrence in Europe. An E. faecium VVE clone belonging to a hospital adapted genetic lineage was dispersed into several wards within a university hospital. This clone carried a transferable plasmid harboring a *vanA* gene cluster variant able to escape phenotypic resistance detection routines but rapidly gaining vancomycin resistance through a single genetic event. We demonstrate that an IS*L3*-like element insertion mediated the silenced VanA phenotype, which could be out-selected due to IS*L3* excision events during vancomycin exposure. This finding represents a novel mechanism for converting *vanA*^+^ VVE from susceptible to resistant. Moreover, detection of the *vanA* carrying plasmid in genetically unrelated E. faecium, as well as in one E. faecalis isolate, strongly points to *in vivo* horizontal transfer events. We provide substantial molecular evidence through PFGE clonality, similarity pattern of *vanA* clusters and presence of similar-sized *vanA*-carrying plasmid of the same broad-host-range replicon type. Importantly, all isolates were linked through epidemiological data. However, we acknowledge that WGS data for all isolates would have provided an even stronger evidence for both clonal and plasmid spread in this outbreak.

The *vanA* cluster contained by Tn*1546* or its derivatives is usually located on transferable plasmids, including both broad-host-range Inc18 (pHTβ1 and pIP501-/pRE25-like) and narrow-host-range RepA_N familes (pRUM-, pLG1-, and pAD1-like) and mosaic combination of these (45–48). In the present study, a plasmid belonging to replicon class 1, represented by pIP501, appeared to mediate both intra- and interspecies transfer of the *vanA* cluster *in vivo*. In a previous study investigating the host range of enterococcal *vanA* plasmids (49), intergenus transfer was also detected for class 1 replicons, underlining an even larger potential for spread of vancomycin resistance by this type of plasmid.

IS*L3*, IS*1216*, and IS*1542* have been associated with broad-host-range plasmids and implied to rearrange mobile genetic elements in enterococci (50). The insertions of IS*1542* upstream of *vanRS* and IS*1216* between *vanX* and *vanY* have been observed by several other groups (23, 24, 29, 51–53) and in many cases have been reported to lead to VanB or VanD phenotype with high-level vancomycin resistance and low-level teicoplanin resistance. If such strains are exposed to teicoplanin over time, the teicoplanin MIC increases, implying IS-mediated genetic rearrangements of the *vanA* cluster.

For the isolates in our study, excision of IS*L3* resulted in expression of the *vanHAX* operon and in high-level vancomycin and teicoplanin resistance. Despite the IS*1542* insertion, a low-level expression of *vanRS* was observed. Phenotypic data from others indicate that loss of VanR leads to complete inactivation of *vanHAX* (19, 25) and that the loss of VanS leads to constitutive expression of *vanHAX* by putative autophosphorylation of VanR (16, 54). Activation of *vanHAX* in the absence of *vanRS* has only been seen by introduction of IS elements upstream of *vanHAX* providing accessory promoters (31). Taken together, this suggest a functional VanRS activation loop of the VVE in our study.

The outbreak investigation was initialized by two cases of *in vivo* switching from vancomycin-susceptible to vancomycin-resistant E. faecium, isolated from the patients before and after treatment with vancomycin. We also observed resistance development during *in vitro* exposure of vancomycin. Above the clinical breakpoint levels (8 μg/ml), resistance occurred within 2 days, or not at all. In the few cases where growth did not occur, we speculate that vancomycin depleted viable bacteria before mutations had the possibility to arise. The observations that bacteria were able to survive for several days during subclinical breakpoint exposure to glycopeptides (2 μg/ml) before growing support this hypothesis and highlights the risk for *in vivo* development caused by subinhibitory concentrations. Under these conditions, presumably providing a wider window in which advantageous mutations could occur, we observed a variety of mutations enabling both high-level and low-level glycopeptide resistance in revertants.

Acquisition of VanA and subsequent *vanA* expression poses a significant initial decrease in fitness for E. faecium or S. aureus, as assessed by several groups (55–57). This fitness cost is then alleviated by unspecified changes within the bacteria if they are allowed to grow in several hundred generations (55). According to Foucault et al. (57, 58), fitness loss is correlated to the expression of vancomycin resistance genes. In our experiments, the expression levels of *vanRS* and *vanHAX* were lowered in both *vanA* cluster variants due to IS insertions. A wide range of Tn*1546* variants with IS insertions have been detected in clinical isolates (23, 59, 60). It might be speculated that IS element insertions in the *vanA* gene cluster result in a functional fitness gain in the absence of glycopeptides.

The nature of the VVE isolates showing altered resistance phenotypes potentiates serious clinical problems both regarding detection, surveillance, horizontal spread of vancomycin resistance and, most severely, the risk of treatment failure. Since detection of VRE usually depends on phenotypic characterization prior to genotypic analysis, VVE would be overlooked. Future phenotypic resistance detection methods giving susceptibility answers within hours after sampling (61) probably have even greater risk of missing out on these rearranged *vanA* gene clusters, since the mutation events reverting to vancomycin resistance take longer to appear. Currently, the overall prevalence of VVE cannot be accounted for. We conclude that VVE have a considerable potential to spread undetected and recommend that enterocooci should be tested by both genotypic and phenotypic methods.

## Supplementary Material

Supplemental material
