# Supplementary material for: A Silenced vanA Gene Cluster on a Transferable Plasmid Caused an Outbreak of Vancomycin-Variable Enterococci
Source: Antimicrob Agents Chemother. 2016 Jun 20;60(7):4119–27. doi: 10.1128/AAC.00286-16 (PMC4914660; doi:10.1128/AAC.00286-16)
Supplement: Supplemental material [file supp_60_7_4119__index.html]

A Silenced vanA Gene Cluster on a Transferable Plasmid Caused an Outbreak of Vancomycin-Variable Enterococci — Supplemental material 

# A Silenced *vanA* Gene Cluster on a Transferable Plasmid Caused an Outbreak of Vancomycin-Variable Enterococci

## Supplemental material

- Supplemental file 1 -

  Supplemental Methods, Table S1-S3, and Fig. S1-S3

  PDF, 1.8M
