# Supplementary material for: A Silenced vanA Gene Cluster on a Transferable Plasmid Caused an Outbreak of Vancomycin-Variable Enterococci
Source: Antimicrob Agents Chemother. 2016 Jun 20;60(7):4119–27. doi: 10.1128/AAC.00286-16 (PMC4914660; doi:10.1128/AAC.00286-16)
Supplement: Supplemental material [file AAC.00286-16_zac007165315so1.pdf]

Supplementary note

SUPPLEMENTAL METHODS

Clinical and screening sample processing. Urine cultures were plated on blood agar (Oxoid, Basingstoke, United Kingdom) and CPSE agar (ChromID CPS Elite, bioMerieux, Marcy L'Etoile, France); blood cultures on BD BACTEC TM Plus + Aerobic/F and BD BACTEC TM Plus + Anaerobic/F bottles (Beckton Dickinson and Company, Sparks, MD); tissue samples and abscesses on blood agar, chocolate agar (GCagar base (Oxoid, Basingstoke, United Kingdom) and Hemoglobin (BBLTM, Beckton Dickinson and Company), FAA agar (Oxoid, Basingstoke, United Kingdom) and fastidious anaerobic broth (LABM, Heywood, United Kingdom); and wound swabs on blood and chocolate agar.

Pulse-Field Gel Electrophoresis. One colony of *E. faecium* from blood agar was incubated overnight at 37°C in 5 mL Todd Hewitt broth. Then, 500 µl of the suspension was centrifuged at 3300 rcf for 2 min, washed with 1 ml TEN-buffer (Tris-EDTA-NaCl, pH 7.5) buffer, centrifuged and dissolved in 250 µl EC-buffer (6 mM Tris-Hcl, EDTA, NaCl, 0.5% Brij 58, 0.2% deoxycholate, 0.5% sarcosyl) and 250 µl 2 % Low Melting Point Agarose (VWR, 15517-014, Invitrogen). 80-100 µl of this suspension was poured into block molds. Agarose embedded cells were lysed with a mix of 3 ml EC buffer, 5 µl Mutanolysin (10000 U/ml) (Sigma-Aldrich, M9901-5K), 50 µl RNase 1 mg/ml (Ribonuclease A, Sigma-Aldrich, R4875) and 100 µl Lysozyme 20 mg/ml (Sigma-Aldrich, R6876) at 37°C for five hours, and thereafter with a mix of 4 ml EC buffer and 100 µl Proteinase K 20 mg/ml (Qiagen, 19133) at 50°C overnight. Slices of the plugs (ca 1.5 mm) were digested with 1 µl SmaI (20000 U/ml)(Sigma-Aldrich, R0141S), 10 µl CutSmart® buffer and 89 µl dH_2O for 2h at 300 rpm at 25°C. *Staphylococcus aureus* NCTC 8325 was used as a size marker on each run. The fragments were separated using CHEF-XA mapper (Bio-Rad) with 1 % Pulsed field certified agarose (Bio-Rad, 162-0137) in 0.5x TBE buffer, temperature 14°C , voltage of 6 V/cm , run time 12h + 10h, initial switch time 5-15 s and final switch time 15-30 s. The gels were stained with GelRed™ (Biotium, Hayward, USA).

TABLE S1. Origins of screening and clinical samples in *vanA* screening.

Sample type	Total number of *vanA* PCRs	*vanA* PCR		Culture positive *vanA* containing	
		+	-	VVE-S	VVE-R
Screening sample	14883				
Rectum/faeces/perineum	14631	81	14550	31	14
Catheter insertion site	144	0	144		
Urine	43	0	43		
Drainage fluid	12	0	12		
Wound	44	0	44		
Respiratory	9	0	9		
E. faecium from culture	275				
Urine	157	5	152	2	3
Blood culture	33	2	31	1	1
Tissue sample	12	0	12		
Ascites / Peritoneal fluid/Drainage fluid/aspirate	42	0	42		
Respiratory	5	0	5		
Abscess	13	1	12		1
Wound	8	4	4	1	3
Miscellaneous**	5	0	5		
Total	15158	93	15065	35	22

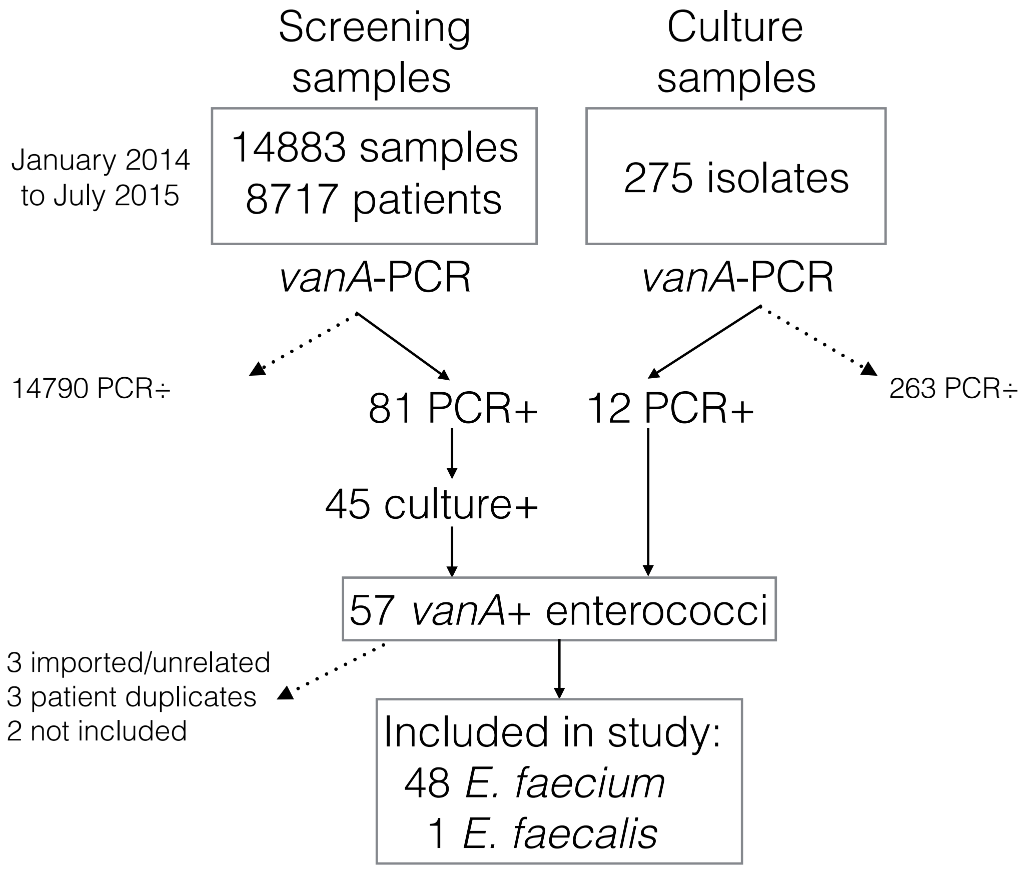

FIG S1. Flow diagram showing which isolates were included (whole arrows) or excluded (dotted arrows) in further caracterization.

Table S2. Primers and probes used in this study

	Forward primer	Reverse primer	Probe
**qPCR**			
vanA Screening	ATGGCAAGTCAGGTGAAGATGG	TCCACCTCGCCAACAACTAACG	CCGGTGGCAGCTACGTTTACCTATCCTG
vanRS	TGTGGCGATTGTCATTAGTATTCTTATTCTATG	AATGCCGGTATTTATCTCGTCAAAGT	TCGCGTCATGCTTTC
vanHAX	CTACTCCCGCCTTTTGGGTTATTAA	CCGGCTTAACAAAAACAGGATAGGT	CCGGCCTATCATCTTT
gdh	AGCCGCTTTCGTTCCGATAAA	GCCTTGAAGATTGGGAAAGAGTGTT	AACGCCAGTCAAATTG
***vanA* cluster closure**			
orf2vanR	TCGGATGAGACAACGTGAAG	GATAGTAAGGCCGCTTGTGC	
vanRS	AAATTGCCGATTTGGTTGAA	TCGCTGGAAGCTCTACCCTA	
IS*1542_closure*	TCTCTTCTGCGGACTTCCTG	CAAGCCGATGACTATGAACG	
vanSH	AATTATTGTTCAGCATGGAGGGCAG	TTTGGCCTTGGATTCCGACAC	
ISL3_closure	ATGTGCGAACCAACTGACTT	CGGAATTGGGCATCGTTCTT	
vanHAX	CATCCCCGTTTTATTTGGTG	AGCTCACCCGTGTCTAATCG	
vanXY	GCTATTTTGATTTCCCCGTTA	GCCACCCTTTACAGCATCAT	
vanYZ	CCTGTTCGCCAAGAAAGTGT	ATGGGTACGGTAAACGAGCA	
**Linkage to plasmid backbone**			
pVVE_1	TGTTGGAGGCTTTCTTGGAC	TTTGCTTTTACCTGGCTTGG	
pVVE_2	CCAAGCCAGGTAAAAGCAAA	CGTTTTAGGGCGTTCTGCTA	
pVVE_3	AAAGGCGCTGACAAATTCTT	CGTGTTTGC-GCTTCTTGATA	
pVVE_4	GTAATCCGAAGCGGTTTTCA	AACATTTGGACTGAATCTGATAAAA	
pVVE_5	TCCAAGGAATCATTGAAATCG	ATGGCAAGCCAGAAACAAAA	
pVVE_6	TTCACGTTGCCAAAAATCAA	AGCCGGTTAAGTGGTCAAAC	
**Southern Hybridization**			
vanA	GTTGCAATACTGTTTGGGGG	CCCCTTTAACGCTAATACGATCAA	
pIP501	TCGCTCAATCACTACCAAGC	CTTGAACGAGTAAAGCCCTT	

TABLE S3. Demographic data and resistance characterization of isolates in study

ID and demographic data			Phylogeny		Vancomycin resistance and IS-elements in cluster (1)					Characterization of *in vitro* generated revertants (1)						Other resistance determinants		
ID	Sampling date	Ward	PFGE type	MLST	MIC µg/ml (Etest)	Pheno-type	IS*L3*	IS*1216*	IS*1542*	*vancomycin concent-ration*	Van rev	IS*L3*	IS*1216*	IS*1542*	MIC µg/ml van/tei (2)	Phenotypic resistance (3)	Genotypic resistance determinants (4)	SRA Accession number
Case1VVE-S	July-13	W1	I	ST 203	1	S	P	P	P	2 µg/ml	Yes	*	P	P	32/4	amp R	AG(*aac(6')-aph(2''), aac(6')-Ii, ant(6)-Ia*), MA(*ermB, msrC*), TET(*tetU, tetM*), *cat*	SRX1496320
										8 µg/ml	Yes	A	P	P	>256/256			
Case1VVE-R	August-13	W1	I	ST 203	>256	R	A	P	P							amp R	AG(*aac(6')-Ii, ant(6)-Ia*), MA(*ermB, msrC*), TET(*tetM*), *cat*	SRX1513504
Case2VVE-S	January-14	W2	I	ST 203	0,75	S	P	P	P	2 µg/ml	Yes	A	P	*	>256/12	amp R	AG(*aac(6')-aph(2''), aac(6')-Ii, ant(6)-Ia*), MA(*ermB, msrC*), TET(*tetU, tetM*), *cat*	SRX1513507
										8 µg/ml	No							
Case2VVE-R	January-14	W2	I	ST 203	>256	R	A	P	P							amp R	AG(*aac(6')-aph(2''), aac(6')-Ii, ant(6)-Ia*), MA(*ermB, msrC*), TET(*tetU, tetM*), *cat*	SRX1513508
Screen1VVE-S	February-14	W3	I	ST 203	1	S	P	P	P	2 µg/ml	Yes	A	P	*	>256/16	amp R	AG(*aac(6')-aph(2''), aac(6')-Ii, ant(6)-Ia*), MA(*ermB, msrC*), TET(*tetU, tetM*), *cat*	SRX1513509
										8 µg/ml	Yes	A	P	*	>256/24			
Screen2VVE-S	February-14	W3	I	ST 203	1	S	P	P	P	2 µg/ml	Yes	A	P	P	>256/256	amp R	AG(*aac(6')-aph(2''), aac(6')-Ii, ant(6)-Ia*), MA(*ermB, msrC*), TET(*tetU, tetM*), *cat*	SRX1513510
										8 µg/ml	Yes	A	P	P	>256/>256			
Case3VVE-R	December-13	W4	I	ST 203	>256	R	A	P	P							amp R		
Case4VVE-R	December-13	W5	I	ST 203	>256	R	A	P	P							amp R		
Case5VVE-R	January-14	W6	II	ST 78	>256	R	A	P	P							amp R		
Screen3VVE-S	February-14	W3	I		1	S	P	P	P	8 µg/ml	Yes	A	P	P		amp R		
Screen4VVE-R	February-14	W2	I		>256	R	*	P	P							amp R		
Screen5VVE-S	February-14	W7	I		2	S	P	P	*	8 µg/ml	Yes	*	P	*		amp R		
Screen6VVE-S	February-14	W1	I		2	S	P	P	P	8 µg/ml	Yes	A	P	P		amp R		
Screen7VVE-R	February-14	W8	III		>256	R	A	P	P							amp R		
Screen8VVE-S	February-14	W9	I		2	S	P	P	P	8 µg/ml	Yes	A	P	P		amp R		
Case6VVE-R	February-14	W8	I		>256	R	A	P	P							amp R		
Screen9VVE-R	March-14	W10	I		>256	R	A	P	P							amp R		
Screen10VVE-R	March-14	W8	I		>256	R	A	P	P							amp R		
Screen11VVE-S	March-14	W3	I		1	S	P	P	*	8 µg/ml	Yes	A	*	*		amp R		
Screen12VVE-S	March-14	W3	I		2	S	P	P	*	8 µg/ml	Yes	A	*	A		amp R		
Screen13VVE-S	March-14	W11	I		2	S	P	P	*	8 µg/ml	Yes	A	*	*		amp R		
Screen14VVE-S	March-14	W5	I		2	S	P	P	*	8 µg/ml	Yes	A	P	*		amp R		
Screen15VVE-S	March-14	W5	I		1	S	P	P	*	8 µg/ml	Yes	A	P	*		amp R		
Screen16VVE-S	March-14	W5	I		2	S	P	P	*	8 µg/ml	Yes	A	*	A		amp R		
Screen17VVE-S	March-14	W5	I		2	S	P	A	*	8 µg/ml	Yes	A	P	*		amp R		
Screen18VVE-S	March-14	W2	I		2	S	P	P	*	8 µg/ml	Yes	?	*	*		amp R		
Screen19VVE-S	April-14	W3	I		1	S	P	P	*	8 µg/ml	Yes	A	P	*		amp R		
Screen20VVE-S	April-14	W5	I		1	S	P	P	*	8 µg/ml	Yes	A	P	*		amp R		
Screen21VVE-S	April-14	W12	I		1	S	P	P	P	8 µg/ml	Yes	A	P	P		amp R		
Screen22VVE-S	April-14	W3	I		1	S	P	P	P	8 µg/ml	Yes	A/P	P	P		amp R		
Screen23VVE-R	April-14	W13	IV		>256	R	A	P	P							amp R		
Screen24VVE-S	April-14	W3	I		1	S	P	P	P	8 µg/ml	Yes	A	P	P		amp R		
Screen25VVE-R	May-14	W13	IV		>256	R	A	P	P							amp R		
Screen26VVE-S	May-14	W14	I		2	S	P	P	P	8 µg/ml	Yes	A	P	P		amp R		
Screen27VVE-S	May-14	W2	I		1	S	P	P	P	8 µg/ml	Yes	A	P	P		amp R		
Screen28VVE-S	May-14	W2	I		1	S	P	P	P	8 µg/ml	Yes	A	P	P		amp R		
Screen29VVE-R	May-14	W15	I		>256	R	A	P	P							amp R		
Screen30VVE-S	May-14	W5	I		2	S	P	P	P	8 µg/ml	Yes	A	P	P		amp R		
Screen31VVE-S	June-14	W3	I		2	S	P	P	P	8 µg/ml	Yes	A	P	P		amp R		
Screen32VVE-S	July-14	W16	I		2	S	P	P	P	8 µg/ml	Yes	A	P	P		amp R		
Screen33VVE-S	July-14	W16	I		2	S	P	P	P	8 µg/ml	Yes	A	P	P		amp R		
Screen34VVE-S	August-14	W6	I		1	S	P	P	P	8 µg/ml	Yes	A	P	P		amp R		
Screen35VVE-S	September-14	W16	I		2	S	P	P	P	8 µg/ml	Yes	A	P	*		amp R		
Screen36VVE-R	September-14	W14	I		>256	R	A	P	P							amp R		
Screen37VVE-S	October-14	W17	I		2	S	P	P	P	8 µg/ml	Yes	*	P	P		amp R		
Screen38VVE-S	January-15	W2	I		1	S	P	P	P	8 µg/ml	Yes	*	P	P		amp R		
Screen39VVE-S	January-15	W2	I		1	S	P	P	P	8 µg/ml	Yes	A	P	P		amp R		
Screen40VVE-S	February-15	W17	I		2	S	P	P	P	8 µg/ml	Yes	A	P	P		amp R		
Screen41VVEfs-S	May-15	W18			1	S	P	P	P	8 µg/ml	Yes	A	P	P				

1: A=absent from gene cluster. P: Present in gene cluster. * indicates no PCR product. ? if multiple products in PCR
2: van=vancomycin, tei=teicoplanin
2: phenotypic tests with disk diffusion method: Ampicillin (Amp), Linezolid, Tigecyclin. Linezolid and tigecyclin resistance was not found in any isolate.
3: Genotypic search with Resfinder (https://cge.cbs.dtu.dk/services/ResFinder/). AG=aminoglycosides, MA=macrolides, TET=tetracyclin, *cat*=chloramphenicol

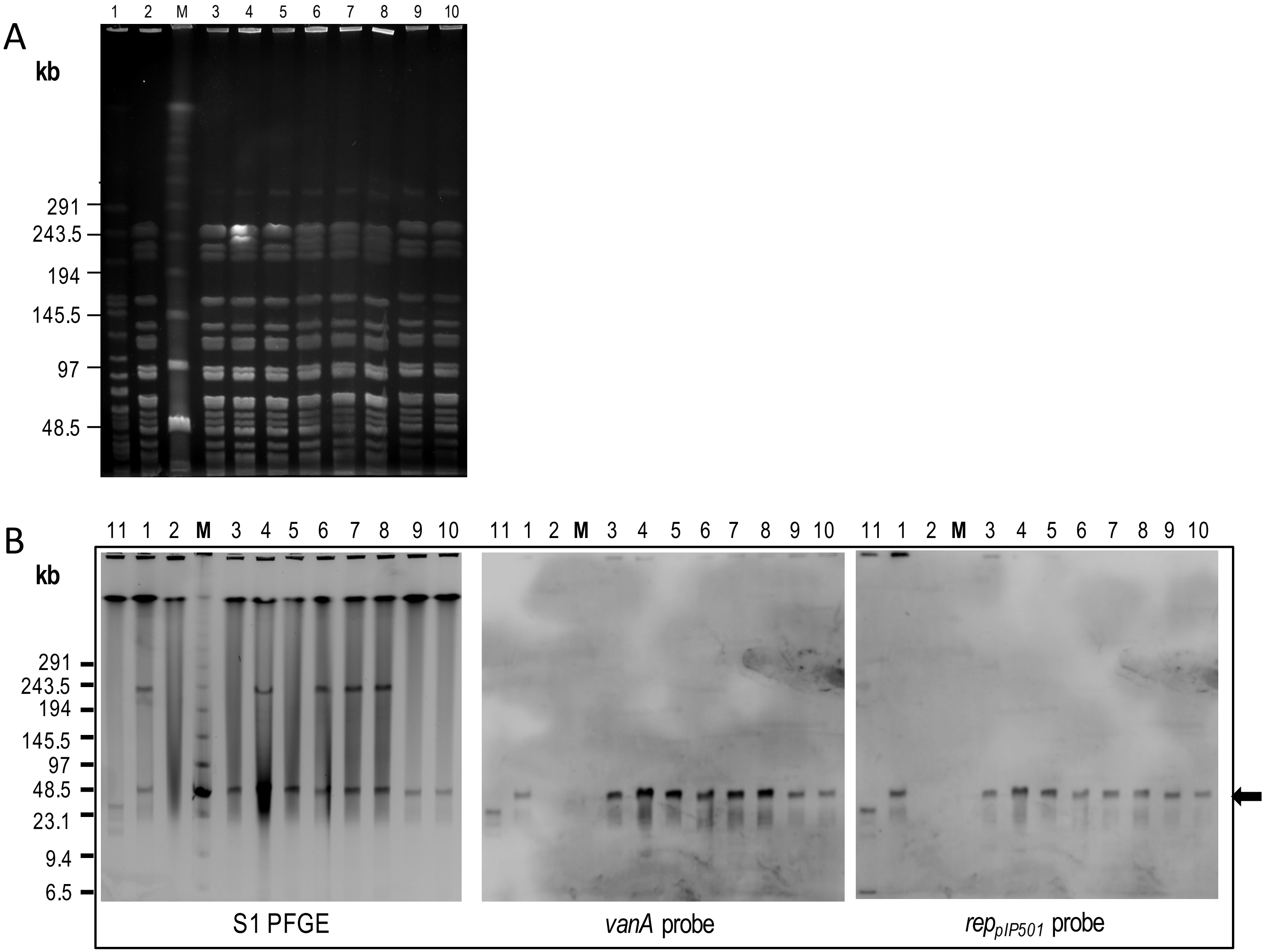

FIG S2. Evidence of horizontal gene transfer between donor Case1VVE-S and Case1VVE-R and recipient 64/3. A) SmaI restriction analyses of transconjugants from Case1VVE-S and Case1VVE-R donors. B) Co-hybridization of *vanA* and plasmid-specific probe for transconjugants obtained by filter mating of Case1VVE-S and Case1VVE-R donors using chloramphenicol (chl) or vancomycin (van) selection by PFGE with S1-nuclease restriction and Southern Hybridization. Lane 11: BM4147 *vanA*$^{+}$ control. 1: Case1VVE-S. 2: 64/3. 3-5: Case1VVE-S x 64/3 chl transconjugants. 6-8: Case1VVE-R x 64/3 chl transconjugants. 9-10: Case1VVE-R x 64/3 van transconjugants.

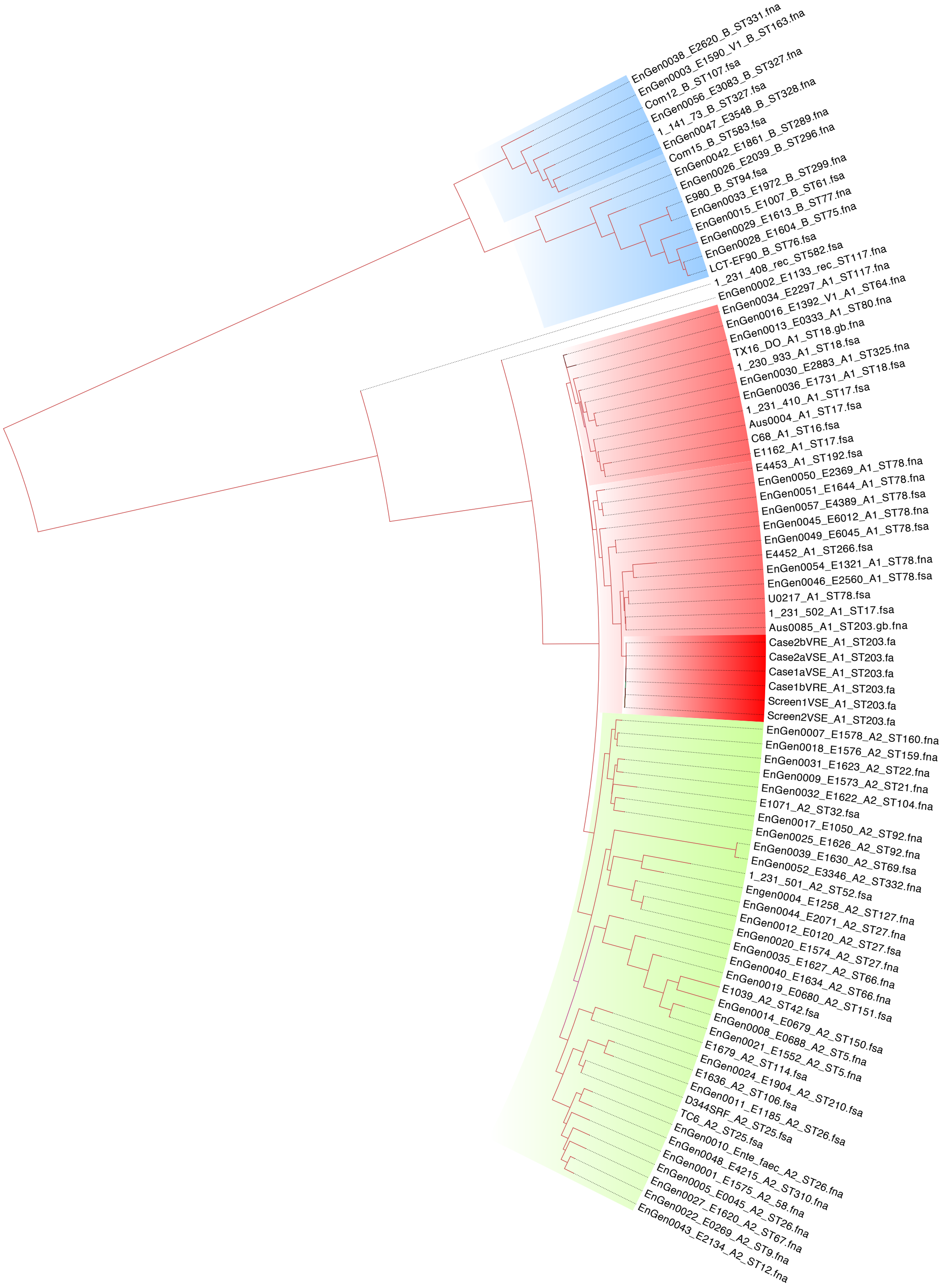

FIG S3. Parsnp (1) WGS phylogeny of *E. faecium* showing livestock-associated strains (green), commensal strains (blue) and nosocomial-associated strains (light red) respectively baptized clade A1, A2 and B by Lebreton *et al.*(2). Our WGS strains (dark red) all belong to ST203 and clade A1. The isolates from Norway are indistinguishable and belonged to the A1 clade. The figure was created with FigTree.

1. **Treangen TJ**, **Ondov BD**, **Koren S**, **Phillippy AM**. 2014. The Harvest suite for rapid core-genome alignment and visualization of thousands of intraspecific microbial genomes. Genome Biol **15**:524.

2. **Lebreton F**, **van Schaik W**, **McGuire AM**, **Godfrey P**, **Griggs A**, **Mazumdar V**, **Corander J**, **Cheng L**, **Saif S**, **Young S**, **Zeng Q**, **Wortman J**, **Birren B**, **Willems RJL**, **Earl AM**, **Gilmore MS**, **Manson McGuire A**, **Godfrey P**, **Griggs A**, **Mazumdar V**, **Corander J**, **Cheng L**, **Saif S**, **Young S**, **Zeng Q**, **Wortman J**, **Birren B**, **Willems RJL**, **Earl AM**, **Gilmore MS**. 2013. Emergence of epidemic multidrug-resistant *Enterococcus faecium* from animal and commensal strains. MBio, 2013/08/22 ed. **4**:e00534–13–e00534–13.
